# A web-based lifestyle intervention program for Chinese college students: study protocol and baseline characteristics of a randomized placebo-controlled trial

**DOI:** 10.1186/s12889-019-7438-1

**Published:** 2019-08-13

**Authors:** Wei Liang, Yan Ping Duan, Bo Rui Shang, Yan Ping Wang, Chun Hu, Sonia Lippke

**Affiliations:** 1 Department of Sport and Physical Education, Faculty of Social Science, Hong Kong Baptist University, Kowloon Tong, Hong Kong, China; 2 Department of Kinesiology, Hebei Institute of Physical Education, Shijiazhuang, China; 3 The National Physical Fitness Lab, Hubei Institute of Sport Science, Wuhan, China; 4 Department of Psychology & Methods, Jacobs University Bremen, Bremen, Germany

**Keywords:** Lifestyle, Physical activity, Healthy diet, eHealth, College students

## Abstract

**Background:**

This study aimed to describe the design and present the baseline characteristics of a web-based lifestyle intervention program, which comprises of sequentially and simultaneously delivered intervention modules targeting physical activity (PA) and fruit and vegetable consumption (FVC) in Chinese college students.

**Methods:**

The study adopted a randomized placebo-controlled trial, using the Health Action Process Approach (HAPA) and the Compensatory Carry-Over Action Model (CCAM) as the theoretical backdrops. 556 Chinese college students participated in the 8-week web-based lifestyle intervention program. All eligible participants were randomly assigned to one of four groups: 1) the PA-first arm which received a 4-week intervention addressing PA followed by a 4-week intervention addressing FVC; 2) the FVC-first arm which received a 4-week intervention addressing FVC followed by a 4-week intervention addressing PA; 3) the PA + FVC simultaneous arm that received an 8-week intervention addressing both PA and FVC at the same time; and 4) the placebo-control arm that received 8 weeks of general health information, which is not relevant for changing actual PA and FVC behaviors. Data collection includes four time-points: at the beginning and end of the intervention, and a 3-month and 12-month follow-up after the intervention.

**Results:**

At baseline, 41.7% of participants were male and 58.3% were female. 41.0% of the participants did not meet the standard PA-recommendations, while 69.6% did not adhere to the standard FVC-recommendations. In total, only 19.6% of participants met both PA and FVC recommendations. Baseline characteristics across the four groups had no significant differences (all *P* = .17–.99), indicating successful randomization.

**Conclusions:**

The preliminary results indicate a high prevalence of unhealthy lifestyles in college students in China, which further supports the need for web-based health intervention programs. This is also the first study that examines the comparative effectiveness of simultaneously and sequentially delivered lifestyle interventions in the Chinese population. These findings may contribute to the creation of future web-based health behavior change interventions.

**Trial registration:**

ClinicalTrails.gov: NCT03627949, 14 August, 2018.

**Electronic supplementary material:**

The online version of this article (10.1186/s12889-019-7438-1) contains supplementary material, which is available to authorized users.

## Background

Physical activity (PA) and fruit-vegetable consumption (FVC) are critical health-related lifestyle behaviors, which substantially contribute to the reduction of non-communicable diseases (NCDs), such as cardiovascular disease, certain types of cancer, type-II diabetes and obesity [1–3]. However, an overwhelming body of evidence has shown that a large proportion of the adult population, especially college students, does not meet the recommendations for physical activity and FVC [4–7]. In China, studies have indicated that about 40% of Chinese college students do not achieve the WHO recommendations for PA (at least 150 min of accumulated PA with moderate and vigorous intensities a week), and less than half of this population adheres to the minimum FVC recommendation (at least five servings of fruit and vegetables per day) [6, 7]. Evidence has demonstrated that unhealthy lifestyle habits in college students can persist after their graduation, and lead to not only short-term (e.g., decrease of immune capacity, body fitness, cognitive functioning, sleep quality and recovery from daily hassles) but also long-term negative outcomes, such as an increased risk for non-communicable diseases such as cardiovascular diseases, obesity and bone or joint complications, and an increased risk for mental health problems such as depression and anxiety [8, 9]. Adopting a healthy lifestyle in college students is essential to prevent short and long- term negative consequences.

This decade has witnessed a burgeoning application of healthy lifestyle interventions targeting both physical activity and dietary behavior in various populations [10, 11]. Compared to interventions focusing only on one certain behavior (e.g., PA or FVC), interventions that target multiple health behaviors (e.g., PA and FVC) consider the synergistic effects or additive effects of diverse health behaviors and have strong potential to advance health promotion, augment health benefits, and reduce healthcare costs [12–15]. For college students, the effectiveness of internet-delivered PA and FVC interventions has been supported by a growing body of evidence [4, 16]. For instance, Greene et al. (2012) developed a 10-week online nutrition and PA intervention program for college students and indicated a significant improvement in PA and FVC after the 10-week intervention and 15-month follow-up in the experimental group compared to the control group (Eta^2^ = .01–.08, *p* < .05) [17]. However, the majority of existing studies have been conducted in Western countries, while research on Chinese college students is comparatively limited.

In addition to this, the timing sequence of treatment delivery in health behavior interventions has yet to be investigated. This question refers to whether or not it is more effective to intervene on diverse behaviors *simultaneously* or change one behavior after the other (*sequentially*) [18, 19]. Opinions and hypotheses regarding this approach differ greatly. One view posited that addressing multiple health behaviors simultaneously is better because it might carry additive and synergistic effects [20, 21]. Another view argued that the simultaneous approach may overburden individuals, as it requires too much self-regulatory effort in the short-term [22]. Therefore, a sequential rather than simultaneous approach could be more suitable [23]. A recent review of six RCTs in adults compared the effectiveness of the two approaches in promoting diverse health behavior change (e.g., PA, dietary behavior, alcohol and smoking) and found both approaches may be equally efficacious, with no significant differences [24]. However, there remains little evidence regarding the comparative effectiveness of simultaneous and sequential approaches, particularly for PA and FVC among college students.

The internet has been widely used to deliver health behavior interventions [4, 25, 26]. For college students who are major users of such technology, web-based interventions seem more attractive, convenient, and cost-effective than other traditional intervention types [27]. Web-based health behavior interventions allow for individualized feedback and follow-ups based on specific characteristics and previously provided information of the users [28]. However, such web-based interventions targeting both PA and FVC in Chinese college students are still scarce [29]. Additionally, in our previous pilot study examining the effects of a web-based intervention on improving PA and FVC behaviors of college students, only one timing sequence of intervention delivery (PA-first) was tested [24]. Moreover, there was a high dropout rate (71.2%) in the pilot study. The current study extended the intervention delivery modules (PA-first, FVC-first, and PA + FVC simultaneously) from the previous study. Furthermore, a refined design and improved function of the website platform was managed to ensure recruitment, engagement and retention of the participants. To the best of our knowledge, this study was the first to examine the effectiveness of web-based interventions targeting both PA and FVC with different delivery time sequences in Chinese college students.

In light of the aforementioned research gaps, this paper aimed to describe the design and to present the baseline characteristics of a web-based lifestyle intervention program, comprising of sequentially and simultaneously delivered treatments targeting PA and FVC. The hypothesis was that 1) participants in the intervention groups would improve their health behaviors, social-cognitive predictors of behaviors change, and health outcomes relative to a placebo-control condition; 2) participants in the sequential arms would show more short-term improvement in these dependent variables (at the end of intervention), while those in the simultaneous arm would show more long-term improvement (3 months and 12 months after the intervention). The results of this study impact the development of future web-based health intervention programs among college students.

## Methods

### Study design

**Fig. 1:**
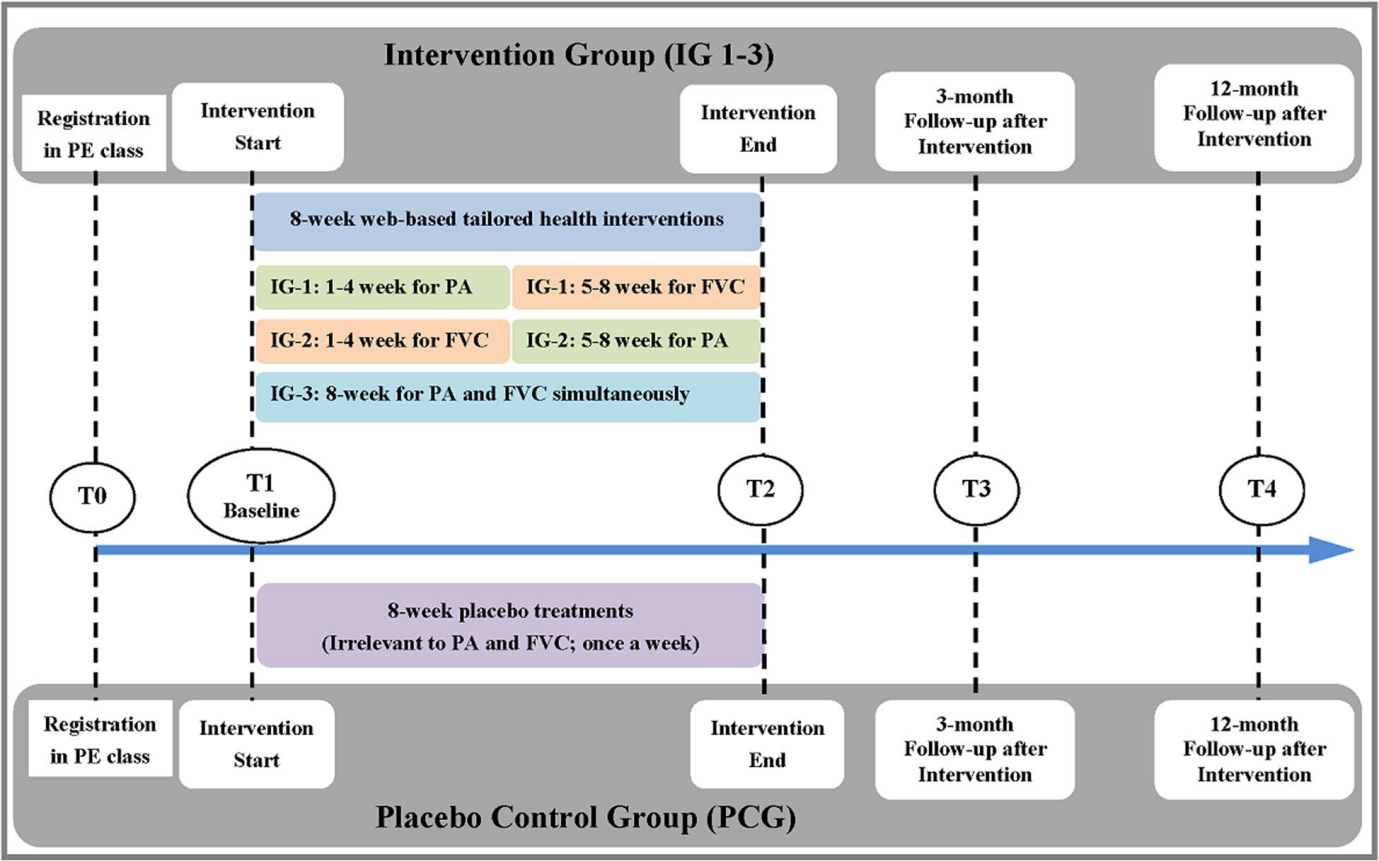
Study process of the web-based healthy lifestyle intervention program

This study was a randomized placebo-controlled trial, with a standard four-arm parallel and double-blinded design. The study consisted of three intervention groups (IGs) and one placebo-control group (PCG). IG-1 (PA-first arm) first addressed PA, then FVC. IG-2 (FVC-first arm) first addressed FVC, then PA. IG-3 (Simultaneous arm) addressed PA and FVC at the same time. The PCG was provided with 8-week placebo treatments, which were irrelevant to PA or FVC. The participant recruitment and online registration were conducted 1 month before the intervention start (T0), during which only sociodemographic information was collected. All participants were asked to complete questionnaires at four time-points, including baseline (at the beginning of intervention (T1), the end of 8-week intervention (T2), 3-month follow-up after intervention (T3), and 12-month follow-up after intervention (T4) (see Fig. 1).

### Participant recruitment and randomization

Participants were recruited from one university in the central region of China. The recruitment information was verbally advertised to the college students by their PE lecturers in their first mandatory physical education (PE) course. In China, all college students, by law^1^ are required to take PE courses. Therefore, it is feasible and advantageous to recruit participants by means of PE classes, as each PE class consists of students from different departments independent of their motivation. The nature and purpose of this program was explained to the PE lecturers (in a train-the-trainer set-up). The students were informed that there would be a health learning program, in which they could obtain health-related knowledge and earn 50 RMB (US $7.5) cash as an incentive if they participate in the study and complete the 4-wave data collection. Students were also asked to complete the online health sessions independently and not to consult with others about the contents of the health sessions. The group allocation was blinded for both students and PE lecturers. Once students expressed their interest to the PE lecturers, they were provided with the study consent inform, and were invited to complete the online registration. After registering and screening for eligibility, all qualified participants were allocated with equal probability (in a 1:1:1:1 ratio) to one of the four groups. Assignment to the four conditions was randomized and stratified for PE class type (e.g., dancing, qigong, table tennis, yoga, and Taichi). The randomization was conducted on the first page of the online health session with a permutated block design (block size was 8), according to the randomization block list generated from the backend management system of the website platform.

A randomization check indicated that there were no significant differences across the four groups at baseline with respect to the age (*F*_(3, 552)_ = 1.13, *P* = .34), gender (*χ*^2^ = 4.79, *P* = .19), study year (*χ*^2^ = 8.21, *P* = .51), and relationship status (*χ*^2^ = 1.15, *P* = .76). Thus, the randomization was successful.

### Eligibility criteria

College students (≥18 years old) were eligible to participate in the study if they (1) were not collegiate athletes or vegetarians; (2) had no restrictions to physical mobility (e.g., heart diseases) or fruit-vegetable intake (fruit allergies or diabetes); and (3) were able to use a computer and a mobile phone, and had access to the internet on a regular basis.

### Sample size calculation

In this study, the required sample size was estimated using G*Power 3.1 software. It was hypothesized that the three intervention groups would significantly increase PA and FVC relative to the control group at T2, T3 and T4. Based on the previous study [30], a maximum effect size of 0.45 on PA (Cohen’s d) was assumed. As such, 79 participants were required per group to provide 80% power with alpha of 0.05 to test the hypothesis. After factoring in drop out, around 25% of participants were added per group [28]. A total of 424 participants (106 per group) were determined to be recruited.

### Intervention

The intervention was guided by the theoretical backdrop of the Health Action Process Approach (HAPA) [31] and the Compensatory Carry-Over Action Model (CCAM) [15]. The HAPA model suggests that the process of behavior change can be divided into two distinctive phases: the motivational and volitional stages, during which individuals may experience a dynamic process from the formation of intention to the performance of behavior. At the beginning of behavioral change, called the motivational phase, an individual develops the intention to perform a specific health behavior. During this stage, specific crucial factors such as action self-efficacy, outcome expectancies, and risk perceptions can collectively contribute to intention formation. Subsequently, once a “good intention” is initiated, the individual enters the volitional phase. During this process, the individual benefits the most from planning (e.g., action planning and coping planning), which can bridge the gap between intention and action. Before the behavior becomes a stable habit, maintenance and recovery self-efficacies and other resources (e.g., social support) play irreplaceable roles in maintaining the behavior change and avoiding relapse. To guide the simultaneous and sequential intervention components, the CCAM was employed to support the transfer of one behavior to another.

**Fig. 2:**
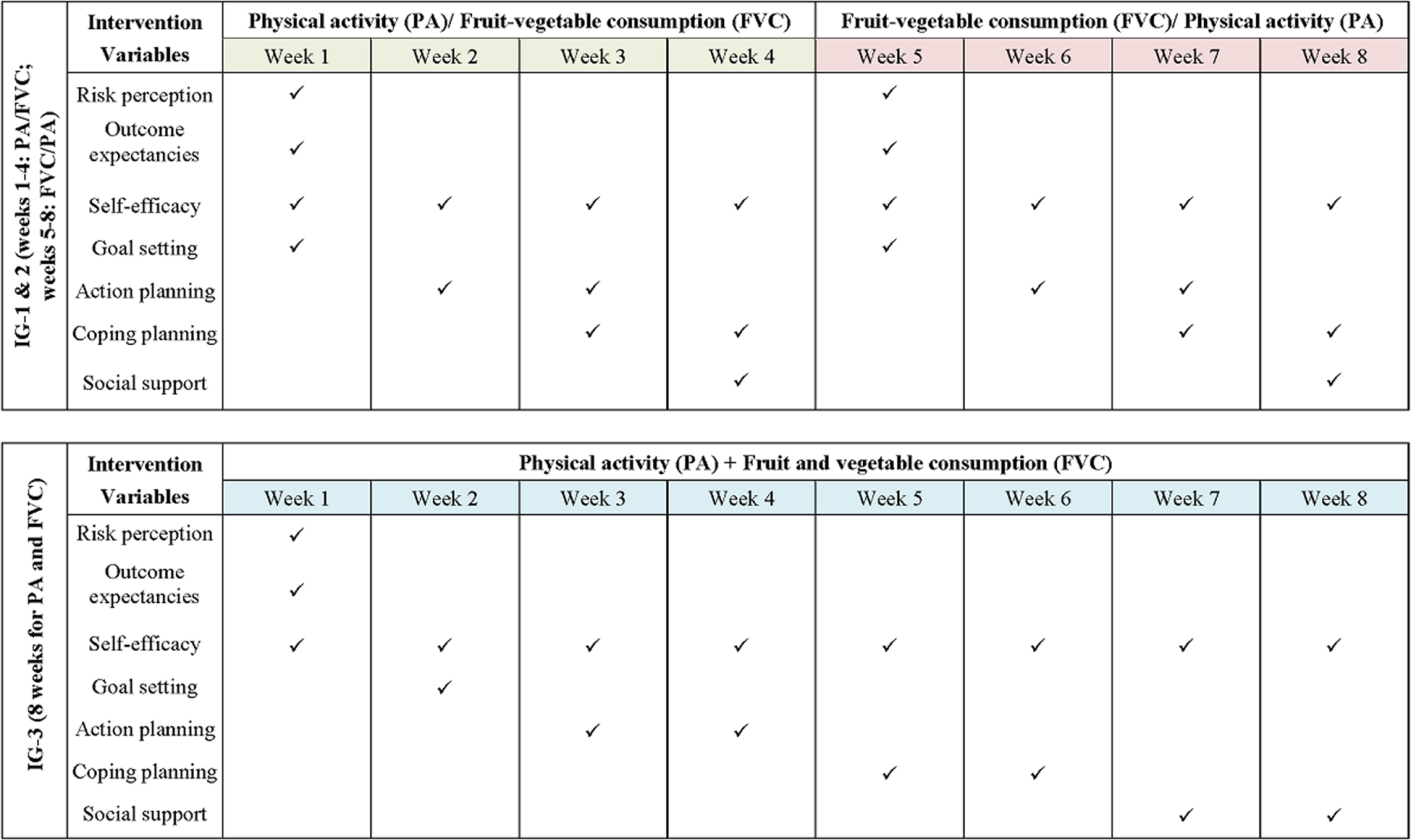
Intervention variables of the web-based lifestyle intervention program

The duration of the web-based lifestyle interventions was 8 weeks (see Fig. 1) [29, 32, 33]. For the PA-first arm, the content included first a 4-week treatment addressing PA, and a subsequent 4-week treatment addressing FVC. For the FVC-first arm, only the sequence of intervention delivery was changed, with FVC addressed first followed by PA. The simultaneous arm had the same amount of treatments for PA and FVC as the sequential arms, but addressed these two behaviors simultaneously for 8 weeks (see Fig. 2). The treatments for the three interventions groups focused on improving social-cognitive variables related to PA and FVC behavior change, including risk perception, outcome expectancies, goal setting, self-efficacy-beliefs, action planning, coping planning and social support (see Additional file 1).

**Fig. 3:**
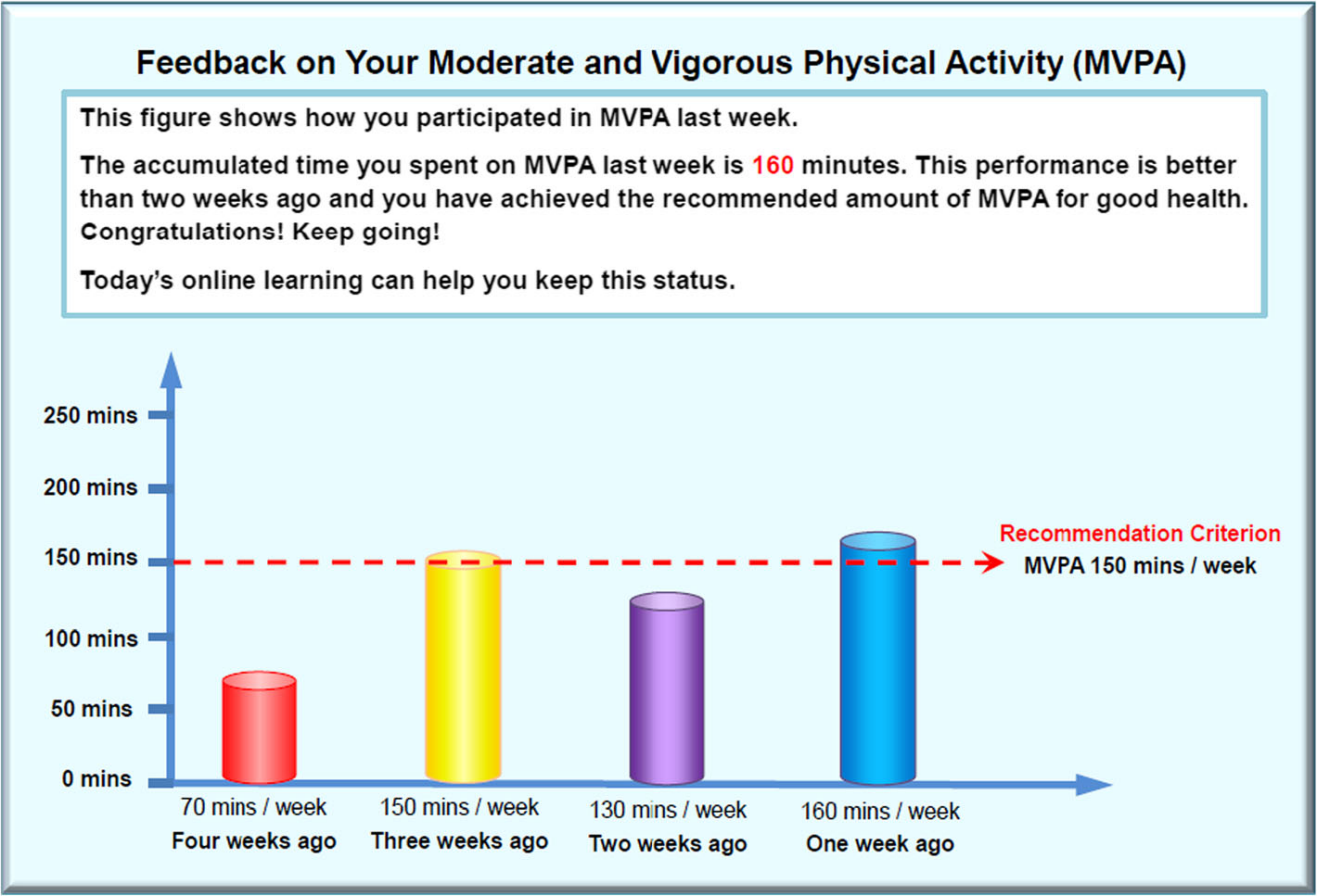
Example of individualized and normative feedbacks on physical activity in the web-based lifestyle intervention program (translated from Mandarin)

Additionally, in order to facilitate the implementation and maintenance of health behavior, behavior change techniques (BCT; e.g., provide information about health consequences, provide instruction on how to perform the behavior, barrier identification, relapse prevention, prompt review of behavioral goals, facilitating social comparison; see Additional file 2) were used in the intervention sessions based on the 93-item BCT taxonomy v1 [34]. Participants were provided with two types of feedback, including individualized feedback on past behavior, and normative feedback on whether the participants met the criterion regarding the behavioral recommendations (see Fig. 3). Furthermore, in order to maximize retention rate, multiple reminder strategies were implemented. For example, the PE lecturers reminded all to click the weekly hyperlink of the health session and follow the online instructions. Meanwhile, SMS and *WeChat* (a prevalent mobile social media application in China) messages were distributed to the participants weekly, prior to each intervention session in order to remind students to participate in the weekly intervention (e.g., Dear student, the new health session will start tomorrow. We kindly remind you to engage in this week’s health session by clicking the hyperlink in your computer which has been sent to you via WeChat this morning. Have a nice day!).

In order to avoid the social desirability and expectation/Hawthorne effects, participants in the control group received placebo treatments which appeared in all respects to be identical to the intervention in the IGs (e.g., the intervention duration and procedure), but lacked the critical ingredients of PA and FVC treatment. In particular, participants were provided with general health information which was irrelevant for changing actual PA and FVC behaviors, such as an introduction to tourist attractions, tips on acupressure massage, and an introduction to some relaxing music and movies. All interventions were delivered on a newly updated web-site platform, through which all participants in the IGs and PCG were invited to attend the health session once per week. They were informed that the intervention would last for around 20 min per session.

Due to the randomization, only the website system could record the participants’ identity and group allocation. When students logged into the website, the system automatically linked them to the different modules according to their group allocation at baseline. With this technology, both intervention and control participants were blinded with respect to the group allocation and reminders.

### Measurements

**Fig. 4:**
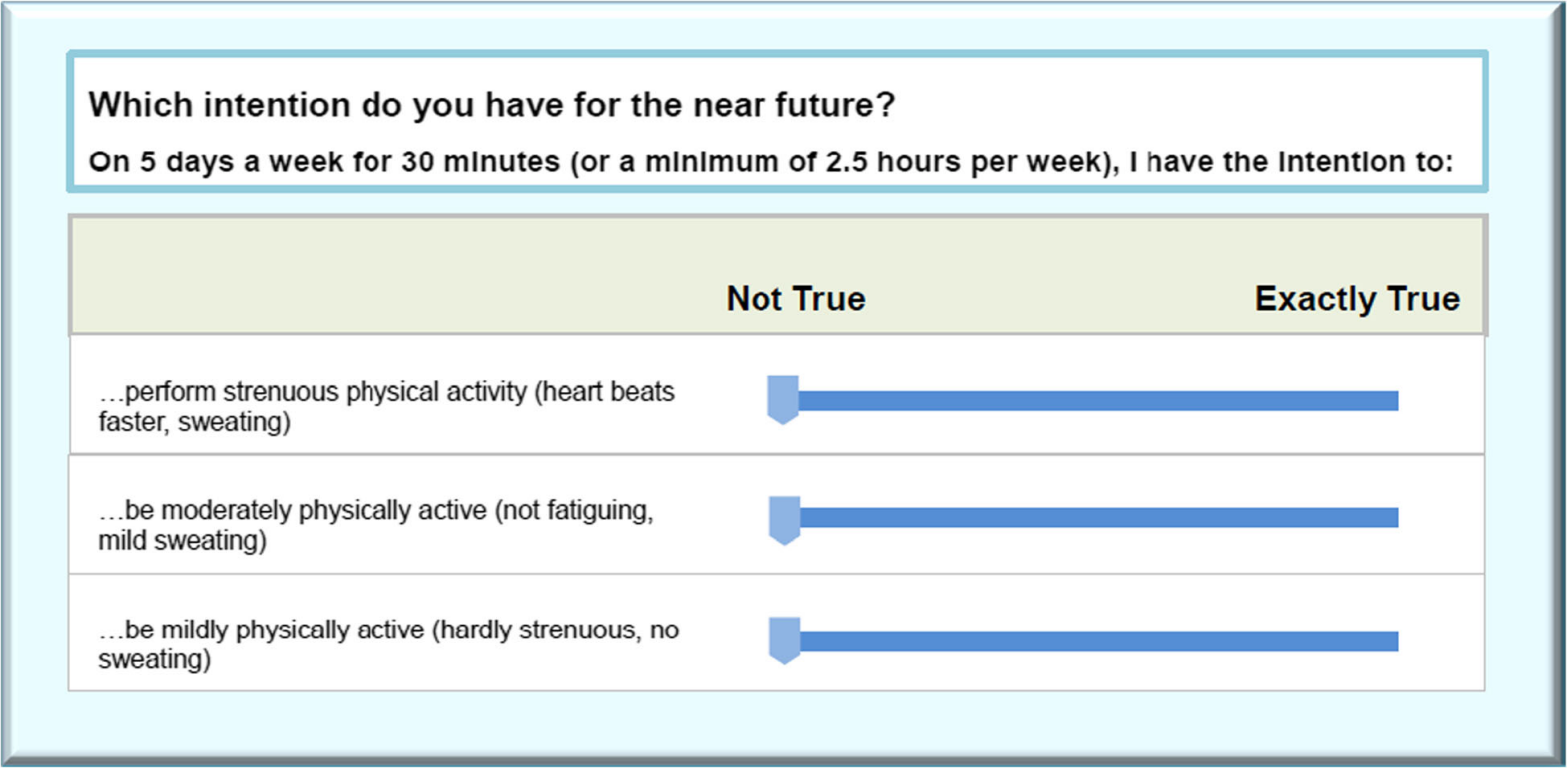
Example of VAS of intention for physical activity in the web-based lifestyle intervention program (translated from Mandarin)

The majority of the items in this study were measured via visual analogue scales (VAS) (example, see Fig. 4). VAS in web-based research has been suggested to be better than traditional Likert-type scales, because of its user friendly appearance and design, more accurate responses, and lower dropout rate. The validity of this has been supported by empirical and theoretical evidence [35].

### Single behavioral and health lifestyle indicators

#### Physical activity (PA)

The weekly amount of PA was measured by the short Chinese version of the International Physical Activity Questionnaire (IPAQ-C) [36]. Participants were asked to estimate the number of days and time spent for vigorous, moderate and walking activities during the past week, with items such as “During the past 7 days, on how many days did you do vigorous physical activities like heavy lifting, digging …” . The total PA score (in minutes/week) for each student was obtained by summing up all questions about PA.

#### Fruit and vegetable consumption (FVC)

Daily FVC was measured with four items, consisting of “raw vegetables”, “fruit”, “raw fruit or vegetable juice”, and “cooked or steamed vegetables” followed by a guide of counting the servings for each type of FVC. For example “last week, how many portions of fruit did you eat per day” [29, 33, 37]. Participants were asked to count the number of portions of fruit and vegetables they consumed on average during a typical day. The total serving of FVC was the sum of each relevant item.

#### Integrated health lifestyle indicator

This study focused not only on the changes of single health behaviors, but also on the improvement of healthy lifestyle as a whole, comprising of both PA and FVC behaviors. Participants were categorized into one of two groups depending on if they meet both PA and FVC recommendations (coding 0 and 1 respectively). The thresholds were based on the WHO recommendations in terms of at least 150 min of accumulated moderate and vigorous PA (MVPA) per week, and at least five servings of fruit and vegetables per day [33, 38, 39].

### Social-cognitive predictors of behavior change

#### Self-efficacy

Self-efficacy was measured by three dimensions including motivational, maintenance and recovery self-efficacies, with the stem “I am certain that …” followed by five items for PA, such as “… I can be physically active a minimum of 5 days a week for 30 minutes, even if it is difficult”, or followed by five items for FVC such as “… I can eat 5 servings of fruit and vegetables per day, even if it is difficult” [29, 33, 40]. Participants were asked to answer all items on a VAS-scale ranging from *don’t agree at all* to *agree completely.*

#### Intention

Intention for PA was assessed with the stem “I intend to perform … at least 30 minutes a day for at least 5 days a week (or at least 150 minutes a week).” followed by three items such as “… strenuous physical activity”, “… moderate physical activity”, and “… mild physical activity”. Regarding FVC, intention was assessed with the stem “I seriously intend to …” followed by three items such as “eat at least 5 portions of fruit and vegetables a day” [29, 33, 41]. The answers were indicated on a VAS-scale ranging from *not true* to *exactly true*.

#### Planning

The variable ‘planning’ was measured by six items, including action planning and coping planning, with three items for each dimension. Action planning for PA was assessed with items such as “… where I will be physically active”, for FVC such as “… at which meals I will eat fruit and vegetables”. Coping planning for PA was assessed with items such as “… what I can do in difficult situations, in order to remain true to my own resolutions”, for FVC such as “… what I can do in difficult situations, in order to remain true to my own resolutions” [29, 31, 33]. Answers were given on a VAS-scale ranging from *don’t agree at all* to *agree completely.*

#### Social support

Perceived social support was assessed with the stem “How do you perceive your environment” followed by three items for PA such as “… People like my friends help me stay physically active”, or followed by three items for FVC such as “… People like my friends help me eat healthily” [29, 33, 42]. Answers were given on a VAS-scale ranging from *don’t agree at all* to *agree completely.*

### Perceived health outcomes

#### Quality of life

Perceived quality of life was measured by the Chinese short version of the WHO Quality of Life-BREF (WHOOQOL-BREF) [29, 33, 43]. The first two items asked about participants’ general quality of life, e.g. “How would you rate your quality of life” (from *very poor* to very *good*), followed by seven items in the physical health sub-domain such as “do you have enough energy for everyday life”, and “how satisfied are you with your ability to perform your daily living activities” with answers of VAS-scale ranging from *very dissatisfied* to *very satisfied*.

#### Depression

Depression was assessed with the Center for Epidemiologic Studies Short Depression Scale (CES-D 10) [29, 33, 44]. The scale was used with the stem “In the past week how often did I feel …” followed by 10 items such as “… I had trouble keeping my mind on what I was doing”. Response options were asked with a VAS-scale ranging from *less than 1 day* to *most or all of the time* (5–7 days).

#### Body mass index (BMI)

Participants were asked to report their body weight (in kg) and body height (in m) for calculating BMI. BMI was calculated by dividing weight (kg) by height squared (m^2^). BMI cut-off points for Asian groups were used to categorize participants into three groups, including underweight if BMI was less than 18.5 kg/m^2^, normal weight if BMI was between 18.5 and 22.9 kg/m^2^, and overweight and obese indicated by a BMI over 23 kg/m^2^ [45].

### Demographic information

Demographic items included gender, age, relationship, study year, and major. All data collection was completed on the newly established website with electronic questionnaires.

### Statistical analysis

The data will be initially treated for missing values using the multiple imputation approach. After examining the distribution of the data, skewed data will be log-transformed and replaced with median values (interquartile range). All analyses will be performed using full intention-to-treat analyses, with scores on dependent variables for dropouts carried over using the multiple imputation method. The Independent samples *t*-tests, Chi-square tests and *F*-tests will be used to analyze differences between drop outs and completers regarding baseline characteristics. Statistical significance will be set at the 5% level (two-tailed).

To test the effectiveness of web-based interventions, linear mixed models with maximum likelihood estimation and generalized linear mixed models with weighted least square estimation will be performed. Regression estimates will be adjusted for participant differences in the number of observations contributing to the mixed models and for variances within subjects [46]. Although gender and age were balanced at baseline, the interaction effects of these variables and treatment should not be omitted [47]. Therefore, fixed effects of the linear mixed models will include testing for time (T1-T4) and treatment (IG-1, IG-2, IG-3 and PCG) effects, adjusted for baseline values, age, gender, and PE class type. In addition, − 2 log likelihood, Akaike and Bayesian information criteria will be used to assess the model fit.

## Results

**Fig. 5:**
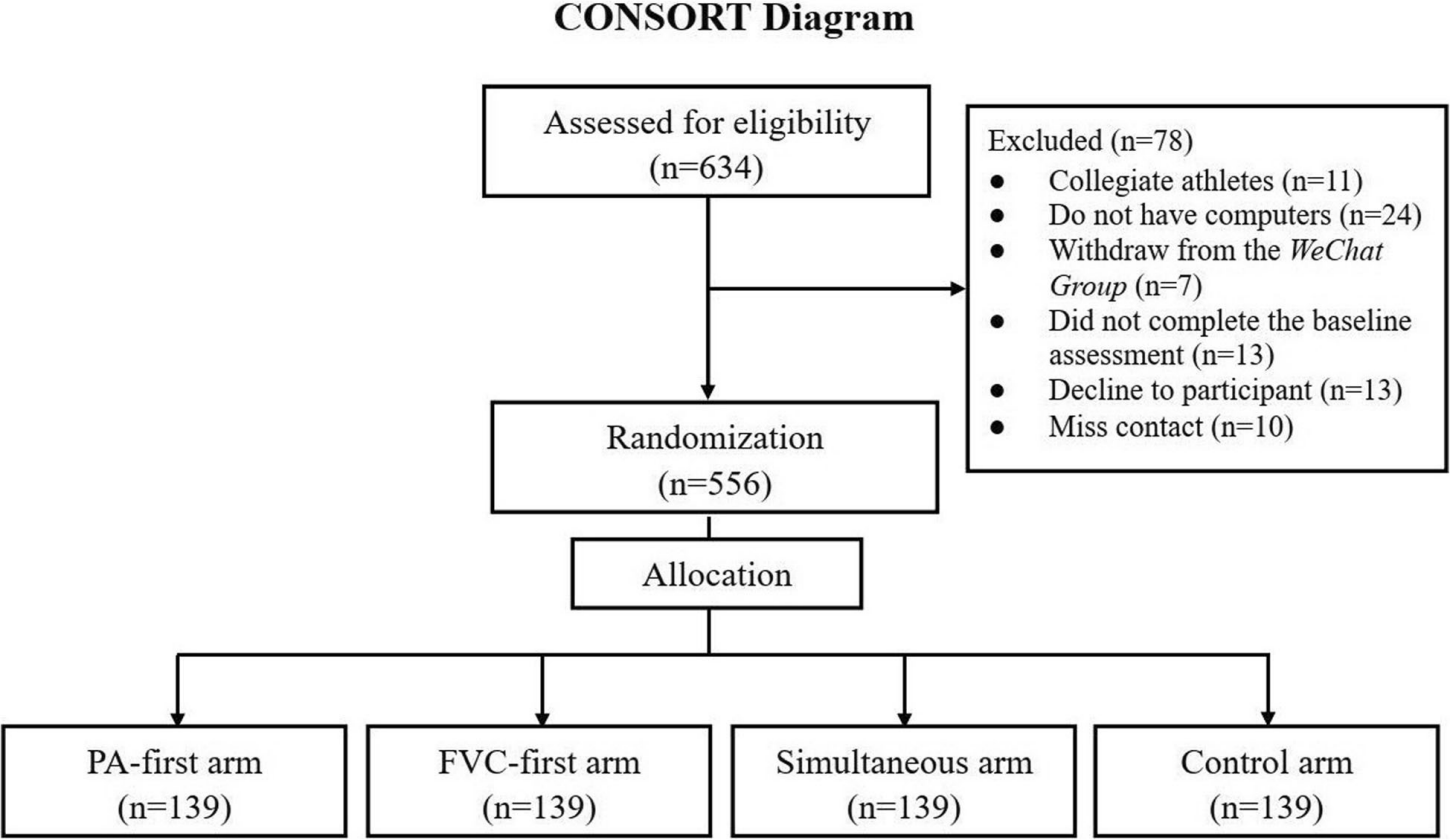
Diagram of participant flow of the web-based lifestyle intervention program

Recruitment for the study was launched in October, 2018. A total of 634 students completed the study registration (14 participants dropped out throughout the registration) and 64 students were excluded after the eligibility screening. Afterwards, all eligible 556 (87.7%) participants were randomly assigned to one of the designated four groups, with 139 students in each group (Fig. 5). The other three waves of the data collection (at the end of intervention, 3-month and 12-month follow-up after intervention) were not included in the current publication.

Of the 556 students who participated in data collection at baseline, 232 were men (41.7%) and 324 were women (58.3%), ranging in age from 18 to 28 years (*Mean* = 20.28 ± 1.28). Most students were freshmen (39.6%) and sophomores (51.8%), while there were only 35 juniors (6.3%) and 13 seniors (2.3%). Among these students, only 57 (10.3%) reported being in a close relationship (having a boyfriend or girlfriend).

Moreover, the descriptive results indicated that 41.0% of the students did not adhere to the MVPA recommendations (150 min/week), while 69.6% of students did not consume at least five servings of fruit and vegetables on average per day. Combining PA and FVC behavior, 30.2% (168/556) of students meet neither the PA nor the FVC recommendations, compared to 50.2% (279/556) who achieved only one recommendation either for PA or for FVC. In total, 109 students met both PA and FVC guidelines, accounting for the smallest proportion (19.6%). The mean BMI was 20.56 ± 2.36 (kg/m^2^), ranging from 16.20 to 27.06 (kg/m^2^). 19.8% of the participants were underweight and 14.4% were overweight. The baseline mean values and standard deviation of dependent variables are displayed in Tables 1 and 2.

**Table 1:** Mean values, standard deviation of the continuous variables among four groups at baseline

	Total (*n* = 556)	IG-1 (*n* = 139)	IG-2 (*n* = 139)	IG-3 (*n* = 139)	PCG (*n* = 139)
MVPA	232.83 (176.52)	234.73 (159.88)	237.79 (181.96)	217.06 (180.76)	241.74 (183.33)
FVC	3.83 (1.76)	3.74 (1.63)	3.92 (1.84)	3.85 (1.86)	3.80 (1.73)
PA INT	2.22 (0.72)	2.21 (0.68)	2.23 (0.72)	2.14 (0.77)	2.30 (0.72)
FVC INT	1.93 (0.76)	1.90 (0.72)	1.92 (0.76)	2.01 (0.81)	1.89 (0.76)
PA SE	2.93 (1.21)	2.96 (1.16)	3.04 (1.22)	2.78 (1.24)	2.94 (1.20)
FVC SE	3.05 (1.37)	2.92 (1.30)	3.03 (1.36)	2.99 (1.46)	3.25 (1.32)
PA Plan	3.01 (1.03)	2.97 (0.97)	3.06 (1.05)	2.87 (1.12)	3.13 (0.95)
FVC Plan	2.81 (1.15)	2.79 (1.10)	2.81 (1.11)	2.72 (1.17)	2.93 (1.22)
PA SS	2.20 (0.91)	2.19 (0.91)	2.21 (0.95)	2.18 (0.95)	2.21 (0.88)
FVC SS	2.37 (0.87)	2.33 (0.82)	2.36 (0.87)	2.34 (0.96)	2.44 (0.82)
Depression	1.94 (0.70)	2.01 (0.72)	1.92 (0.70)	1.90 (0.71)	1.91 (0.69)
QoL	3.42 (0.78)	3.32 (0.80)	3.44 (0.79)	3.43 (0.81)	3.48 (0.74)
BMI	20.56 (2.36)	20.3 (2.19)	20.53 (2.49)	20.83 (2.48)	20.49 (2.25)

Note: *IG* Intervention group, *PCG* Placebo-control group, *MVPA* Weekly amount of moderate and vigorous physical activity (minutes/week), *FVC* Daily portions of fruit-vegetable consumption (portions/day), *INT* Intention, *SE* Self-efficacy, *SS* Social support, *QoL* Quality of life, *BMI* Body mass index (kg/m^2^)

**Table 2:** Crosstab of the categorical variables among four groups at baseline

	IG-1 (*n* = 139)	IG-2 (*n* = 139)	IG-3 (*n* = 139)	PCG (*n* = 139)
BMI (kg/m^2^)				
Underweight (< 18.5)	25	29	31	25
Healthy weight (18.5–22.9)	96	87	87	96
Overweight (≥23)	18	23	21	18
Lifestyle indicator				
Healthy ^a^	108	116	115	108
Unhealthy ^b^	31	23	24	31

Note: *BMI* Body mass index. ^a^ Healthy lifestyle indicates meeting both behavior recommendations (performing ≥150 min of moderate-vigorous physical activity per week and consuming ≥5 portions of fruit and vegetables per day); ^b^ Unhealthy lifestyle indicates not meeting both behavior recommendations (performing ≥150 min of moderate-vigorous physical activity per week and consuming ≥5 portions of fruit and vegetables per day)

## Discussion

This was the first study to design and evaluate the comparative effectiveness of sequentially and simultaneously delivered interventions on lifestyle change in the Chinese population. The baseline findings provided a prerequisite for the subsequent examination of the study hypotheses and also revealed that physical inactivity and inadequate fruit-vegetable consumption are highly prevalent in Chinese college students. This is similar to investigations with Western students [5, 6]. To help college students improve their health status and adopt healthy lifestyles, effective web-based lifestyle intervention programs are necessary and web-based approaches have strong potential.

Given the benefits of tailored multiple lifestyle behaviors interventions, there is still a need for further research regarding various aspects of such interventions. This study protocol provides a research paradigm of exploring more efficacious timing sequence of delivering interventions targeting PA and FVC behaviors in the Chinese population. Moreover, it can be applied not only to PA and FVC promotion, but can also be used as a reference when designing interventions targeting other healthy lifestyle behaviors (e.g., alcohol and smoking cessation).

To improve future research, some limitations of this protocol need to be addressed. First, the system with the PE course in China is ideal for such a study design. However, spill-over or contamination between students in the IG and CG should be controlled in further studies. In addition, this study was conducted only in one university and more universities should be included in the future to control for biases due to enrollment with the university. In this study, all variables were collected by self-reported measures which may cause recall bias. Therefore, using objective measurements (e.g., accelerometers, height and weight measurements) in addition to subjective measures is desirable in future studies. Despite these limitations, this study makes a valuable contribution to the successful future development of web-based lifestyle intervention programs in college students, and the development of online interventions generally. Further data from the follow-up measurement points may give insights into the actual compared effectiveness of different interventions. This was previously done in European countries and America [30, 48] and now for the first time in Asia. Testing this procedure and intervention in other Asian countries is required to test whether effects are generic for Asia or whether it should be adapted.

## Conclusion

A web-based and theory-based lifestyle intervention program targeting PA and FVC was developed for the Chinese college students. Baseline results of the randomized placebo-controlled trial indicated the prevalence of unhealthy lifestyle of college students in China. This is the first study to compare the effectiveness of interventions with different delivery patterns in Chinese college students as well as in the Asian population.

## Additional files

Additional file 1: Detailed intervention content of the web-based lifestyle intervention program. (PDF 823 kb)

Additional file 2: Behavioral change techniques used in the web-based lifestyle intervention program. (PDF 170 kb)

## Data Availability

The dataset supporting the conclusions of this article will be available from the corresponding author upon reasonable request after data collection is finished.

## Abbreviations

BMI: Body mass index
CCAM: Compensatory Carry-Over Action Model
FVC: Fruit and vegetable consumption
HAPA: Health Action Process Approach
IG: Intervention group
MVPA: Moderate and vigorous physical activity
NCD: Non-communicable disease
PA: Physical activity
PCG: Placebo-control group
QoL: Quality of Life
VAS: Visual analogue scales
WHO: World Health Organization

## Footnotes

1
http://www.moe.gov.cn/s78/A10/moe_918/tnull_8465.html

## References

[1] Dietz WH, Douglas CE, Brownson RC (2016). Chronic disease prevention: tobacco avoidance, physical activity, and nutrition for a healthy start. Jama.

[2] Leatherdale ST, Rynard V (2013). A cross-sectional examination of modifiable risk factors for chronic disease among a nationally representative sample of youth: are Canadian students graduating high school with a failing grade for health?. BMC Public Health.

[3] World Health Organization (2018). World health statistics 2018: monitoring health for the SDGs, sustainable development goals.

[4] Oosterveen E, Tzelepis F, Ashton L, Hutchesson MJ (2017). A systematic review of eHealth behavioral interventions targeting smoking, nutrition, alcohol, physical activity and/or obesity for young adults. Prev Med.

[5] Aceijas C, Waldhäusl S, Lambert N, Cassar S, Bello-Corassa R (2017). Determinants of health-related lifestyles among university students. Perspect Public Health.

[6] Li X, Yang H, Yang F (2018). Influences of campus environment on physical activity participation of college students. J Wuhan Inst Phys Educ (in Chinese).

[7] Dong H, Wang Y (2018). Investigation of dietary behavior and physical activity in university students. China Health Care & Nutr (in Chinese).

[8] Deforche B, VanDyck D, Deliens T, DeBourdeaudhuij I (2015). Changes in weight, physical activity, sedentary behavior and dietary intake during the transition to higher education: a prospective study. Int J Behav Nutr Phys Act.

[9] Friedman HS, Martin LR, Tucker JS, Criqui MH, Kern ML, Reynolds CA (2008). Stability of physical activity across the life span. J Health Psychol.

[10] Vandelanotte C, Müller AM, Short CE, Hingle M, Nathan N, Williams SL (2016). Past, present, and future of eHealth and mHealth research to improve physical activity and dietary behaviors. J Nutr Educ Behav.

[11] Reinwand DA, Crutzen R, Storm V, Wienert J, Kuhlmann T, de Vries H, Lippke S (2016). Generating and predicting high quality action plans to facilitate physical activity and fruit and vegetable consumption: results from an experimental arm of a randomised controlled trial. BMC Public Health.

[12] Prochaska Judith J., Prochaska James O. (2011). A Review of Multiple Health Behavior Change Interventions for Primary Prevention. American Journal of Lifestyle Medicine.

[13] Amato K, Park E, Nigg CR (2016). Prioritizing multiple health behavior change research topics: expert opinions in behavior change science. Transl Behav Med.

[14] Wilson K, Senay I, Durantini M, Sánchez F, Hennessy M, Spring B, Albarracín D (2015). When it comes to lifestyle recommendations, more is sometimes less: a meta-analysis of theoretical assumptions underlying the effectiveness of interventions promoting multiple behavior domain change. Psychol Bull.

[15] Lippke S (2014). Modelling and Supporting Complex Behavior Change related to Obesity and Diabetes Prevention and Management with the Compensatory Carry-Over Action Model. J Diabetes Obes.

[16] Epton T, Norman P, Dadzie AS, Harris PR, Webb TL, Sheeran P, Julious SA, Ciravegna F, Brennan A, Meier PS, Naughton D (2014). A theory-based online health behaviour intervention for new university students (U@ Uni): results from a randomised controlled trial. BMC Public Health.

[17] Greene GW, White AA, Hoerr SL, Lohse B, Schembre SM, Riebe D, Patterson J, Kattelmann KK, Shoff S, Horacek T, Blissmer B (2012). Impact of an online healthful eating and physical activity program for college students. Am J Health Promot.

[18] Fleig L, Küper C, Lippke S, Schwarzer R, Wiedemann AU (2015). Cross-behavior associations and multiple health behavior change: A longitudinal study on physical activity and fruit and vegetable intake. J Health Psychol.

[19] Geller K, Lippke S, Nigg CR (2017). Futuredirections of multiple behavior change research. J Behav Med.

[20] Lippke S, Nigg CR, Maddock JE (2012). Health-promoting and health-risk behaviors: theory-driven analyses of multiple health behavior change in three international samples. Intern J Behav Med.

[21] Södergren M, McNaughton SA, Salmon J, Ball K, Crawford DA (2012). Associations between fruit and vegetable intake, leisure-time physical activity, sitting time and self-rated health among older adults: Cross-sectional data from the WELL study. BMC Public Health.

[22] Fleig L, Kerschreiter R, Schwarzer R, Pomp S, Lippke S (2014). ‘Sticking to a healthy diet is easier for me when I exercise regularly’: cognitive transfer between physical exercise and healthy nutrition. Psychol & Health.

[23] Verplanken B, Melkevik O (2008). Predicting habit: the case of physical exercise. Psychol Sport Exerc.

[24] James E, Freund M, Booth A, Duncan MJ, Johnson N, Short CE (2016). Comparative efficacy of simultaneous versus sequential multiple health behavior change interventions among adults: a systematic review of randomised trials. Prev Med.

[25] Cha SA, Lim SY, Kim KR, Lee EY, Kang B, Choi YH, Yoon KH, Ahn YB, Lee JH, Ko SH (2017). Community-based randomized controlled trial of diabetes prevention study for high-risk individuals of type 2 diabetes: lifestyle intervention using web-based system. BMC Public Health.

[26] Stephani V, Opoku D, Quentin W (2016). A systematic review of randomized controlled trials of mHealth interventions against non-communicable diseases in developing countries. BMC Public Health.

[27] Sarcona A, Kovacs L, Wright J, Williams C (2017). Differences in eating behavior, physical activity, and health-related lifestyle choices between users and nonusers of mobile health apps. Am J Health Educ.

[28] Lustria MLA, Noar SM, Cortese J, VanStee SK, Glueckauf RL, Lee J (2013). A meta-analysis of web-delivered tailored health behavior change interventions. J Health Commun.

[29] Duan YP, Wienert J, Hu C, Si GY, Lippke S (2017). Web-Based Intervention for Physical Activity and Fruit and Vegetable Intake among Chinese University Students: A Randomized Controlled Trial. J Med Internet Res.

[30] Schulz DN, Kremers SP, Vandelanotte C, Schneider F, Candel MJ (2014). Effects of a web-based tailored multiple-lifestyle intervention for adults: A two-year randomized controlled trial comparing sequential and simultaneous delivery modes. J Med Internet Res.

[31] Schwarzer R (2008). Modeling health behavior change: how to predict and modify the adoption and maintenance of health behaviors. Appl Psychol.

[32] Reinwand D, Kuhlmann T, Wienert J, DeVries H, Lippke S (2013). Designing a theory-and evidence-based tailored eHealth rehabilitation aftercare program in Germany and the Netherlands: study protocol. BMC Public Health.

[33] Duan YP, Liang W, Guo L, Wienert J, Si GY, Lippke S (2018). Evaluation of a web-based intervention for multiple health behavior changes in patients with coronary heart disease in home-based rehabilitation: pilot randomized controlled trial. J Med Internet Res.

[34] Michie S, Richardson M, Johnston M, Abraham C, Francis J, Hardeman W, Eccles MP, Cane J, Wood CE (2013). The behavior change technique taxonomy (v1) of 93 hierarchically clustered techniques: building an international consensus for the reporting of behavior change interventions. Ann Behav Med.

[35] Kuhlmann T, Reips UD, Wienert J, Lippke S (2016). Using visual analogue scales in eHealth: non-response effects in a lifestyle intervention. J Med Internet Res.

[36] Macfarlane DJ, Lee CC, Ho EY, Chan KL, Chan DT (2007). Reliability and validity of the Chinese version of IPAQ (short, last 7 days). J Sci Med Sport.

[37] Rafferty AP, Anderson JV, McGee HB, Miller CE (2002). A healthy diet indicator: quantifying compliance with the dietary guidelines using the BRFSS. Prev Med.

[38] World Health Organization (2004). Fruit and Vegetables for Health. Report of a Joint FAO/WHOWorkshop.

[39] World Health Organization (2010). Global recommendations on physical activity for health.

[40] Luszczynska A, Sutton S (2006). Physical activity after cardiac rehabilitation: evidence that different types of self-efficacy are important in maintainers and relapsers. Rehabil Psychol.

[41] Lippke S, Wiedemann AU, Ziegelmann JP, Reuter T, Schwarzer R (2009). Self-efficacy moderates the mediation of intentions into behavior via plans. Am J Health Behav.

[42] Jackson J, Lippke S, Gray CD (2011). Stage-specific prediction of physical activity in orthopaedic patients after rehabilitation treatment. Int J Sport Psychol.

[43] Yao G, Wu CH (2009). Similarities and differences among the Taiwan, China, and Hong-Kong versions of the WHOQOL questionnaire. Soc Indic Res.

[44] Rankin SH, Galbraith ME, Johnson S (1993). Reliability and validity data for a Chinese translation of the Center for Epidemiological Studies-Depression. Psychol Rep.

[45] Who EC (2004). Appropriate body-mass index for Asian populations and its implications for policy and intervention strategies. Lancet (London, England).

[46] Raudenbush SW, Bryk AS. Hierarchical linear models: applications and data analysis methods. Thousand Oaks: Sage; 2002.

[47] Peterson RL, Tran M, Koffel J, Stovitz SD (2017). Statistical testing of baseline differences in sports medicine RCTs: a systematic evaluation. BMJ Open Sport Exerc Med.

[48] King AC, Castro CM, Buman MP, Hekler EB, Urizar GG, Ahn DK (2013). Behavioral impacts of sequentially versus simultaneously delivered dietary plus physical activity interventions: the CALM trial. Ann Behav Med.

