# Supplementary material for: A web-based lifestyle intervention program for Chinese college students: study protocol and baseline characteristics of a randomized placebo-controlled trial
Source: BMC Public Health. 2019 Aug 13;19:1097. doi: 10.1186/s12889-019-7438-1 (PMC6692951; doi:10.1186/s12889-019-7438-1)
Supplement: Supplementary file 1 — Detailed intervention content of the web-based lifestyle intervention program. (PDF 823 kb) [file 12889_2019_7438_MOESM1_ESM.pdf]

IG-1 & 2: weeks 1-4 for PA/FVC; weeks 5-8 for FVC/PA		**Physical activity (PA)/ Fruit-vegetable consumption (FVC)**				**Fruit-vegetable consumption (FVC)/ Physical activity (PA)**			
		Week 1	Week 2	Week 3	Week 4	Week 5	Week 6	Week 7	Week 8
	**Intervention Content**	1.Informing about the risk of inactive behavior/ unhealthy diet<br>2.Introducing and prompting the benefits of PA/FVC<br>3.Setting goals for PA/FVC and general health<br>4.Encouraging students to build confidence in PA/FVC	1.Learning how to make specific action plans for PA/FVC<br>2.Providing instruction on how, when and where to perform what type of PA/FVC<br>3.Taking examples of successful cases about action plan execution<br>4.Prompting review of health goals<br>5.Providing encouragement for performing PA/FVC	1.Reviewing the execution of action plans; adjusting action plans<br>2.Finding barriers or difficulties in plan execution; setting coping plans<br>3.Taking examples of successful cases about health behavior adherence to PA/FVC<br>4.Providing encouragement for performing PA/FVC	1.Reviewing the execution of coping plans; adjusting coping plans<br>2. Prompting perceived social support for PA/FVC from significant others. If no support, learn how to seek support (e.g., discuss with roommates or friends about support)<br>3.Taking examples of successful cases about behavior relapse prevention for PA/FVC<br>4.Providing encouragement for performing PA/FVC	1.Informing about the risk of unhealthy diet/ inactive behavior<br>2.Introducing and prompting the benefits of FVC/PA<br>3.Setting goals for FVC/PA and general health<br>4.Encouraging students to build confidence in FVC/PA	1.Learning how to make specific action plans for FVC/PA<br>2.Providing instruction on how, when and where to perform what type of FVC/PA<br>3.Taking examples of successful cases about action plan execution<br>4.Prompting review of health goals<br>5.Providing encouragement for performing FVC/PA	1.Reviewing the execution of action plans; adjusting action plans<br>2.Finding barriers or difficulties with plan execution; setting coping plans<br>3.Taking examples of successful cases about health behavior adherence to FVC/PA<br>4.Providing encouragement for performing FVC/PA	1.Reviewing the execution of coping plans; adjusting coping plans<br>2. Prompting perceived social support for FVC/PA from significant others. If no support, learn how to seek support (e.g., discuss with roommates or friends about support)<br>3.Taking examples of successful cases about behavior relapse prevention for FVC/PA<br>4.Providing encouragement for performing FVC/PA

**IG-3: 8-weeks for PA and FVC**	**Intervention Content**	**Physical activity (PA) + Fruit and vegetable consumption (FVC)**							
		Week 1	Week 2	Week 3	Week 4	Week 5	Week 6	Week 7	Week 8
		1.Informing about the risk of inactive behavior and unhealthy diet<br>2.Introducing and prompting the benefits of PA and FVC<br>3.Encouraging students to build confidence in PA and FVC	1.Setting goals for PA and FVC, as well as general health<br>2.Prompting feedback on PA/FVC performance<br>3.Encouraging students to build confidence in PA and FVC	1.Learning how to make specific action plans for PA and FVC<br>2.Providing instruction on how, when and where to perform what type of PA and FVC<br>3.Taking examples of successful cases about action plan execution<br>4.Prompting review of health goals<br>5.Providing encouragement for performing PA and FVC	1.Reviewing the execution of action plans; adjusting action plans<br>2.Taking examples of successful cases about action plan execution<br>3.Providing encouragement for performing PA and FVC	1.Finding barriers or difficulties with plan execution; setting coping plans<br>2.Taking examples of successful cases about health behavior adherence to PA and FVC<br>3.Providing encouragement for performing PA and FVC	1.Reviewing the execution of coping plans; adjusting coping plans<br>2.Taking examples of successful cases about health behavior adherence to PA and FVC<br>3.Providing encouragement for performing PA and FVC	1. Prompting perceived social support for PA and FVC from significant others. If no support, learn how to seek support (e.g., discuss with roommates or friends about support)<br>2.Taking examples of successful cases about behavior relapse prevention for PA and FVC<br>3.Providing encouragement for performing PA and FVC	1. Prompting perceived social support for PA and FVC from significant others. If no support, learn how to seek support (e.g., discuss with roommates or friends about support)<br>2.Taking examples of successful cases about behavior relapse prevention for PA and FVC<br>3.Providing encouragement for performing PA and FVC

**Placebo control group**	**Placebo Treatments**	Leisure tourism		Winter warmth		Emotion regulation		Music & Movie	
		Introduction to five tourist attractions in Hong Kong	Introduction to five tourist attractions in Hong Kong	Five life tips for keeping warm in winter	Acupressure massage skills for keeping warm in winter	The use of three essential oils for relaxation and emotion adjustment	The use of three general psychological skills for regulating emotions	Introduction to three types of light music for relaxation	Introduction to three interesting movies for relaxation
