# Supplementary material for: A web-based lifestyle intervention program for Chinese college students: study protocol and baseline characteristics of a randomized placebo-controlled trial
Source: BMC Public Health. 2019 Aug 13;19:1097. doi: 10.1186/s12889-019-7438-1 (PMC6692951; doi:10.1186/s12889-019-7438-1)
Supplement: Supplementary file 2 — Behavioral change techniques used in the web-based lifestyle intervention program. (PDF 170 kb) [file 12889_2019_7438_MOESM2_ESM.pdf]

Behavior change techniques (BCTs)	Intervention session
➢ Provide information about health consequences 5.1 (e.g., risks of unhealthy behaviors in general with epidemiological data; benefits of health behaviors to the individual)<br>➢ Goal setting 1.1 (e.g., PA and FVC behaviour change)<br>➢ Goal setting 1.3 (e.g., general health, weight management, energy balance)	IG-1&2: Week 1 & 5;<br>IG-3: Week 1 & 2
➢ Action planning 1.4<br>➢ Provide instruction on how to perform the behaviour 4.1	IG-1&2: Week 2, 3, 6 &7;<br>IG-3: Week 3 & 4
➢ Problem solving 1.2 (e.g., barrier identification/relapse prevention/coping planning)<br>➢ Discrepancy between current behavior and goal 1.6	IG-1&2: Week 3, 4, 7 & 8;<br>IG-3: 5 & 6
➢ Social support (unspecified) 3.1	IG-1&2: Week 4 & 8;<br>IG-3: Week 7 & 8
➢ Prompt review of behavioural goals 1.5<br>➢ Prompt review of outcome goals 1.7<br>➢ Prompting feedback on behavior 2.2<br>➢ Facilitate social comparison 6.2 (e.g., take other people's successful examples)<br>➢ Prompt social reward 10.4 (e.g., written encouraging praise)<br>➢ Prompt verbal persuasion about capability 15.1 (e.g., tell the participants that they can successfully perform the wanted behavior)	IG-1&2: Week 2,3,4,6,7,&8;<br>IG-3: Week 2-8
